# Agreement between Different Methods to Measure the Active Drag Coefficient in Front-Crawl Swimming

**DOI:** 10.5114/jhk/159605

**Published:** 2023-01-20

**Authors:** Jorge E. Morais, Tiago M. Barbosa, Nuno D. Garrido, Maria S. Cirilo-Sousa, António J. Silva, Daniel A. Marinho

**Affiliations:** 1 Department of Sports Sciences, Instituto Politécnico de Bragança, Bragança, Portugal.; 2 Research Centre in Sports, Health and Human Development (CIDESD), Covilhã, Portugal.; 3 Department of Sports Sciences, University of Trás-os-Montes and Alto Douro, Vila Real, Portugal.; 4 Department of Physical Education/LABOCINE, Federal University of Paraíba, João Pessoa, Brazil.; 5 Department of Sports Sciences, University of Beira Interior, Covilhã, Portugal.

**Keywords:** swimming, kinetics, force, hydrodynamics, performance

## Abstract

The aim of this study was to analyze the agreement of the active drag coefficient measured through drag and propulsion methods. The sample was composed of 18 swimmers (nine boys: 15.9 ± 0.9 years; nine girls: 15.3 ± 1.2 years) recruited from a national swimming team. The velocity perturbation method was used as the drag measurement system and the Aquanex system as the propulsion system. For both sexes combined, the frontal surface area was 0.1128 ± 0.016 m^2^, swim velocity 1.54 ± 0.13 m.s^-1^, active drag 62.81 ± 11.37 N, propulsion 68.81 ± 12.41 N. The level of the active drag coefficient agreement was calculated based on the mean values comparison, simple linear regression, and Bland Altman plots. The mean data comparison revealed non-significant differences (p > 0.05) between methods to measure the active drag coefficient. Both the linear regression (R^2^ = 0.82, p < 0.001) and Bland Altman plots revealed a very high agreement. The active drag coefficient should be the main outcome used in the interpretation of the swimmers’ hydrodynamic profile, because it is less sensitive to swimming velocity. Coaches and researchers should be aware that the active drag coefficient can also be calculated based on propulsion methods and not just based on drag methods. Thus, the swimming community can now use different equipment to measure the hydrodynamics of their swimmers.

## Introduction

In competitive swimming, swimming velocity is determined by the net balance between propulsive and drag forces acting on swimmers to determine the body’s displacement (i.e., accelerations and decelerations, respectively) (Barbosa et al., 2020):


$$a=\frac{T-D}{m}\text{ (1)}$$

in which a refers to acceleration (m•s^-2^), T is the total propulsion (N), D is the total drag force (N), and m the swimmer’s body mass (kg). Drag is the resistance force to promote displacement in a fluid environment (such as water), and can be calculated based on Newton’s equation as:


$$D=\frac{1}{2}\cdot \rho \cdot \nu^{2}\cdot FSA\cdot C_{D}\text{ (2)}$$

in which D is the drag (N), ρ is the density of water (997 kg•m^-3^), FSA is the swimmer’s frontal surface area (m^2^), v is the swimming velocity (m•s^-1^), and CD is the drag coefficient (dimensionless). There are two types of drag: (1) passive drag, i.e., water resistance over a swimmer being towed through the water without moving body segments, and (2) active drag, i.e., water resistance while swimming (Pendergast et al., 2005). Thus, the latter (i.e., active drag) receives a lot of attention by researchers and coaches since swimmers spend most time of the race performing the swimming stroke (Morais et al., 2019; Veiga and Roig, 2016).

However, the active drag force may not be the most adequate way to understand the hydrodynamic profile of swimmers (the same rational can be used for the passive drag). Drag was found to be proportional to the square of velocity, i.e., an increase in swimming velocity will promote an exponential increase in drag (Toussaint and Beek, 1992). Thus, taking drag as the reference variable for the swimmers’ hydrodynamic profile can lead to misleading assumptions. For instance, when measuring age-group swimmers based on a longitudinal approach, active drag increased significantly (Morais et al., 2014). However, this may not be related to worse swimming technique, but rather to the increase in swimming velocity, which will directly affect active drag (Morais et al., 2014). As it happens in other sports where the fluid resistance plays a key role, such as cycling (Malizia and Blocken, 2021) and motorsports (Katz, 2021), the best way to understand the dynamics of an object is through the drag coefficient. This allows the analysis of the aerodynamic or hydrodynamic effectiveness based on the fluid resistance of an object or subject regardless of its size or velocity.

Besides the drag methods, the active drag coefficient (CDa) can also be calculated through propulsion methods (Havriluk, 2003). Nowadays, there is user-friendly equipment that measure swimmers’ propulsion without any mechanical restrictions (Havriluk et al., 2013; Koga et al., 2020; Lanotte et al., 2018). After obtaining the propulsion generated by the stroke cycle, swimming velocity, and FSA, it is possible to calculate the CDa based on equation (2). However, as far as our understanding goes, evidence on the level of agreement between the CDa measured by drag and propulsion methods is missing. This information is important to understand whether the two types of equipment measure the same phenomenon, i.e., the hydrodynamic profile of the swimmers.

Therefore, the aim of this study was to analyze the agreement of the CDa measurement through drag and propulsion methods. It was hypothesized that there would be a high level of agreement between the different methods of measuring CD_a_.

## Methods

### Participants

Eighteen swimmers (nine boys: 15.9 ± 0.9 years, 70.73 ± 9.10 kg of body mass, 1.79 ± 0.07 m of body height, 1.87 ± 0.09 m of the arm span; FINA points: 590.11 ± 61.81 in the 100 m freestyle event, short course meter swimming pool; nine girls: 15.3 ± 1.2 years, 57.64 ± 6.11 kg of body mass, 1.63 ± 0.07 m of body height, 1.67 ± 0.09 m of the arm span, FINA points: 601.56 ± 73.38 in the 100 m freestyle event, short course meter swimming pool) were recruited to participate in this study. Swimmers were recruited from a national squad that included swimmers participating at international championships and national record holders (Tier 3; McKay et al., 2022). They had more than 5 years of competitive experience and performed six to seven swimming sessions per week, complemented with at least one dry-land strength and conditioning training session per week. Parents or guardians and swimmers signed an informed consent form. All procedures were in accordance with the Declaration of Helsinki regarding human research, and the Polytechnic of Bragança Ethics Board approved the research design (N.º 72/2022).

### Design and Procedures

The velocity perturbation method was used (Kolmogorov and Duplishcheva, 1992) to compute the active drag coefficient based on a drag method (CDa_VPM). Swimmers performed two 25 m maximum front crawl swimming trials with a push-off start after a standardized warm-up. One trial was performed at maximum speed in front crawl and the other at maximum speed in front crawl while towing a hydrodynamic body (i.e., a perturbation device). This hydrodynamic body was attached to the swimmers’ waist with a belt at an 8 m distance (to minimize drafting effects of the perturbation device in the wake of the swimmer) (Kolmogorov and Duplishcheva, 1992).

In each trial, the string of a speedometer (SpeedRT, ApLab, Rome, Italy) was attached to the swimmers’ hip to measure swimming velocity. The speedometer calculated the displacement and velocity of the swimmer at a sampling rate of 100 Hz. Afterwards, it was imported into signal processing software (AcqKnowledge v. 3.9.0, Biopac Systems, Santa Barbara, USA). The signal was handled with a Butterworth 4^th^ order low-pass filter (cut-off: 5 Hz). A video camera (Sony FDRX3000, Japan) was attached to a rail on the edge of the swimming pool. The camera recorded swimmers in a sagittal plane, and it was synchronized to the speed-time data. Velocity was measured between the 11^th^ and the 24^th^ m as reported elsewhere (Morais et al., 2020a).

The CD_a__VPM was computed as:


$$C_{Da\_VPM}=\frac{2\cdot D_{a}}{\rho \cdot FSA\cdot \nu^{2}}\text{ (4)}$$

in which C_Da_VPM_ is the active drag coefficient (dimensionless), Da is the active drag (N), ρ is the density of water (997 kg•m^-3^), FSA is the swimmer’s cross-sectional frontal surface area (m^2^), and v is swimming velocity (m•s^-1^). The FSA was measured by digital photogrammetry. Swimmers were photographed by a digital camera (Alpha 6000, Sony, Tokyo, Japan) in the transverse plane (downwards view) on land simulating the streamlined position. This position is characterized by the arms being fully extended above the head, one hand over the other, fingers also extended close together and head in a neutral position. The FSA was measured from the swimmer’s digital photo on dedicated software (Udruler, AVPSoft, USA) (Morais et al., 2012). Afterwards, a correction was performed based on the FSA variation during the stroke cycle (Morais et al., 2020a).

The propulsion data were acquired concurrently with the drag data (without the perturbation device). As the VPM measured the swimmer’s drag between the 11^th^ and the 24^th^ m, the average propulsion performed during this distance was used for analysis. Force data acquisition equipment Aquanex (Swimming Technology Research, USA) was used to measure the propulsion (*f* = 100 Hz) (Morais et al., 2020b). This system is based on sensors that estimate the in-water force with a measurement error of 0.2%. Such sensors were placed between the third and fourth metacarpals to measure the pressure differential between the palmar and dorsal surfaces. It is assumed that this place is a good proxy of the application point of the thrust vector on the hand (Gourgoulis et al., 2013). At the beginning of each trial, swimmers were asked to keep their hands vertically immersed at a depth of 0.5 m for 10 s to calibrate the system with the hydrostatic pressure values. The sensors and video output were connected to an A/D converter connected to a laptop on the pool deck with Aquanex software (Aquanex v. 4.2 C1211, Richmond, USA) (Morais et al., 2020b). Afterwards, time-force series were imported into signal processing software (AcqKnowledge v. 3.9.0, Biopac Systems, Santa Barbara, USA). The signal was handled with a Butterworth 4^th^ order low-pass filter (cut-off: 5 Hz).

For each dominant and non-dominant arm-pull, the mean propulsion (F_mean_dominant_ and F_mean_non-dominant_, N) was analyzed. The Ftotal_stroke cycle (the total propulsion produced in one full stroke cycle) was calculated as being the sum of the propulsion produced by the dominant and non-dominant upper limbs. Afterwards, the active drag coefficient based on the propulsive force (CD_a_Thrust_) was computed by inverse dynamics based on equation (2). In equation (2) D corresponds to the drag force, which can be assumed equal to the average propulsion for constant swimming velocity (Havriluk, 2003). The FSA values were the same as previously used.

### Statistical Analysis

The Kolmogorov-Smirnov and the Levene’s tests were used to assess the normality and homocedasticity, respectively. The mean plus one standard deviation, and 95% confidence intervals (95CI) were computed as descriptive statistics. The coefficient of variation (CV, %) was calculated between the CDa_VPM and CD_a_pressure sensors_.

The intraclass correlation coefficient (ICC) was measured to establish homogeneity through the fraction or proportion of the total variability of the measurements (Koo and Li, 2016). Afterwards, the comparative analysis between methods included: (1) mean data comparison; (2) simple linear regression between values, and (3) Bland Altman plots (Barbosa et al., 2017). For the mean data comparison, the student’s *t*-test paired samples (*p* ≤ 0.05), the mean difference with 95% confidence intervals (95CI), and the magnitude of the effect size (Cohen’s *d*) were computed. This effect size index was interpreted as: (1) trivial if 0 ≤ d < 0.20; (2) small if 0.20 ≤ *d* < 0.60; (3) moderate if 0.60 ≤ *d* < 1.20; (4) large if 1.20 ≤ *d* < 2.00; (5) very large if 2.00 ≤ *d* < 4.00; (6) nearly perfect if *d* ≥ 4.00 (Hopkins, 2002).

Simple linear regression models between assessment methods (drag vs. propulsion) were computed. Trendline equation, determination coefficient (R^2^), standard error of estimation (SEE), 95% of confidence (95CI), and prediction (95PI) intervals were calculated. As a general rule and qualitative interpretation, the relationship was defined as: very weak if R^2^ < 0.04; weak if 0.04 ≤ R^2^ < 0.16; moderate if 0.16 ≤ R^2^ < 0.49; high if 0.49 ≤ R^2^ 0.81, and very high if 0.81 ≤ R^2^ < 1.0. The Bland Altman analysis included the plot of the mean value of the drag versus the thrust (i.e., propulsion) (Bland and Altman, 1986). A bias of ± 1.96 standard deviation of the difference was adopted as the limit of agreement. For qualitative assessment, it was considered that the analytical modeling data were valid and appropriate if at least 80% of the plots were within the ± 1.96 standard deviation of the difference (95CI).

## Results

Table 1 presents descriptive data for males, females and both sexes grouped together (overall). The CV between the CD_a_VPM_ and CD_a_pressure sensors_ was 6.24%. The ICC between the CD_a_VPM_ and CD_a_pressure sensors_ was ICC = 0.928 (95CI: 0.796 to 0.973). Table 2 presents the *t*-test comparison between methods. The CDa measured with the VPM and with pressure sensors did not present significant differences with a small effect size (Table 2).

**Table 1:** Descriptive data (mean ± one standard deviation, and 95% confidence intervals – 95CI) for all the variables assessed for males, females, and both sexes plotted together.

	Mean ± 1 SD (95CI)		
	Males	Females	Overall
**FSA [m^2^]**	0.1235 ± 0.016 (0.1113 to 0.1358)	0.1020 ± 0.004 (0.0988 to 0.1052)	0.1128 ± 0.016 (0.1049 to 0.1206)
**v [m•s^-1^]**	1.64 ± 0.08 (1.58 to 1.70)	1.43 ± 0.08 (1.37 to 1.49)	1.54 ± 0.13 (1.47 to 1.60)
**Da [N]**	68.30 ± 13.44 (53.68 to 81.19)	57.31 ± 5.13 (56.07 to 65.17)	62.81 ± 11.37 (57.36 to 70.69)
**C_Da_VPM_**	0.41 ± 0.14	0.59 ± 0.09	0.50 ± 0.14
**[dimensionless]**	(0.31 to 0.52)	(0.52 to 0.66)	(0.43 to 0.57)
**F_mean_dominant_ [N]**	39.28 ± 8.07 (33.07 to 45.48)	30.64 ± 3.30 (28.10 to 33.18)	34.96 ± 7.45 (31.25 to 38.67)
**F_mean_non-dominant_ [N]**	36.96 ± 6.72 (31.80 to 42.13)	30.73 ± 3.86 (27.77 to 33.70)	33.85 ± 6.21 (30.76 to 36.93)
**F_total_stroke_ _cycle_ [N]**	76.24 ± 13.63 (65.76 to 86.72)	61.37 ± 4.13 (58.20 to 64.55)	68.81 ± 12.41 (62.64 to 74.98)
**C_Da_pressure_ _sensors_ [dimensionless]**	0.47 ± 0.11 (0.38 to 0.55)	0.59 ± 0.07 (0.54 to 0.65)	0.53 ± 0.11 (0.47 to 0.59)

FSA – frontal surface area; v – swimming velocity; Da – active drag; CDa_VPM – active drag coefficient based on the velocity perturbation method; Fmean_dominant – mean propulsion of the dominant upper-limb; Fmean_non-dominant – mean propulsion of the non-dominant upper-limb; Ftotal_stroke cycle – total propulsion of the full stroke cycle; CDa_pressure sensors – active drag coefficient based on the pressure sensors.

**Table 2:** Comparison between the CDa computed based on the velocity perturbation method and on the pressure sensors method (i.e., propulsion).

	T-test comparison			
	Mean difference (95CI)	*t-test*	*p*	d [descriptor]
**C_Da_VPM_ vs C_Da_pressure sensors_ mensionless**	0.029 −0.002 to 0.	1.95	0.068	0.24 [small]

CD_a_VPM_ – active drag coefficient based on the velocity perturbation method; CD_a_pressure_ sensors – active drag coefficient based on the pressure sensors (i.e., propulsion).

**Figure 1:**
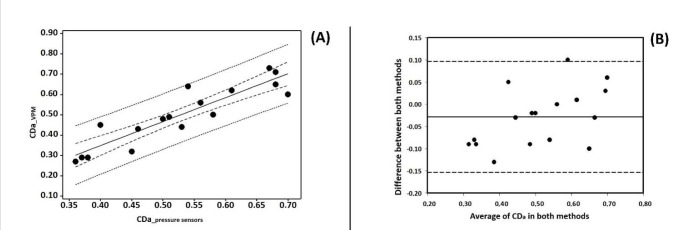
Panel A – linear regression analysis between the active drag coefficient measured with the velocity perturbation method (VPM), and with the pressure sensors. Solid line represents the linear regression trend, dash lines the 95% confidence intervals (95CI), and point lines the 95% prediction intervals (95PI). Panel B – Bland Altman plots of the active drag coefficient measured with the velocity perturbation method (VPM), and with the pressure sensors. CD_a_VPM_ – active drag coefficient based on the velocity perturbation method; CD_a_pressure sensors_ – active drag coefficient based on the pressure sensors.

Figure 1 (Panel A) depicts the linear regression (R^2^ = 0.82, *p* < 0.001, SEE = 0.06) between methods for the CDa with 95CI and 95PI intervals. The relationship was of very high agreement. Figure 1 (Panel B) depicts the Bland Altman analysis, in which only one swimmer was not within the 95CI agreement. Thus, the agreement criterion, according to which more than 80% of the plots must be within 95CI agreement, was accomplished.

## Discussion

This study aimed to analyze the agreement between measuring the CDa based on drag and propulsion methods. The main results indicate that non-significant differences were observed between the methods with a very high relationship and that the Bland Altman criterion was accomplished.

The literature reports four experimental approaches to measure the active drag: (1) the measuring active drag (MAD) system (Hollander et al., 1986); (2) the assistant towing method (ATM) at constant speed (Alcock and Mason, 2007), and at fluctuating speed (Mason et al., 2011); (3) the residual thrust method (MRT) (Narita et al., 2017), and; (4) the velocity perturbation method (VPM) (Kolmogorov and Duplishcheva, 1992). However, the MAD, ATM, and MRT methods are complex, time-consuming, and expensive systems. On the other hand, the VPM method is simple to set up and therefore less time-consuming, inexpensive, and non-invasive, making it more suitable for the assessment of young swimmers.

Indeed, several studies have used the VPM to measure the hydrodynamics of swimmers (Marinho et al., 2010; Morais et al., 2014; Toussaint et al., 2004) as its reliability to measure the swimmers’ active drag has already been shown (Toussaint et al., 2004). Additionally, most studies related to the assessment of active drag using all three remaining methods (MAD, ATM, and MRT systems) do not present data related to CDa (e.g., Formosa et al., 2012; Hazrati et al., 2016; Neiva et al., 2021). Conversely, studies in which the VPM method was used did present the CDa output (Barbosa et al., 2015; Morais et al., 2014).

Regarding propulsion gear, there are wearable systems that measure the swimmers’ propulsion based on user-friendly setups. These systems are mostly based on independent pressure sensors attached to the swimmers’ hands allowing their displacement such as under “free swimming” conditions (Santos et al., 2022; Koga et al., 2020; Lanotte et al., 2018). A study by van Houwelingen et al. (2017) indicated that research about propulsion must focus on determining the best path and the velocity profile of the hand, hand shape(s) throughout the stroke cycle, and the role of the entire swimmer’s body in producing propulsive force. However, this summary was based on numerical studies. There is no evidence about this topic through experimental procedures and thus, it can be argued that there is still no experimental gold standard method for measuring propulsion in swimming.

Moreover, studies that used this kind of equipment to measure swimmers’ propulsion did not report any CDa output (Koga et al., 2020; Morais et al., 2020b). Nonetheless, it can be argued that the focus of such studies was to mainly understand the swimmers’ propulsion (force that promotes forward motion) and not the water resistance acting on the swimmer’s body. It must be pointed out that the drag MRT method evaluates the drag in front-crawl swimming based on the relationship between propulsion and drag (Narita et al., 2017). It acknowledges that the difference between propulsion and drag occurs from changing the flow velocity, i.e., residual thrust (Narita et al., 2017). In this case, this research group did present the CDa output (Narita et al., 2018a). However, as aforementioned, this method does not rely on an exclusively propulsion method.

Data from the present study showed non-significant differences between the CDa based on both methods and a high level of agreement between them. To the best of our knowledge, this is the only study that aimed to understand the agreement between methods for the CD_a_ measurement, specifically between drag and propulsion methods. Based on the present results, it can be confirmed that the CDa can be measured by different methods as they all measure the same phenomenon. Comparison studies were conducted, but only based on drag procedures and for active drag alone (Formosa et al., 2012; Narita et al., 2018b; Toussaint et al., 2004). However, the overall trend reported in the literature is that the active drag output presents significant differences when measured by different methods. For instance, Toussaint et al. (2004) aimed to compare the MAD and the VPM methods. The authors observed that the two methods produced significant differences in the active drag output, however, they recognized that both methods measured the same phenomenon. Others compared the active drag values between the MAD and the ATM methods (Formosa et al., 2012). For the same mean maximum speed both systems differed by 55% in magnitude and the values obtained were significantly different between the two methods (higher in the ATM). The authors argued that the kicking absence in the MAD system might partially explain such a large difference (Formosa et al., 2012). More recently, Narita et al. (2018b) compared the MAD and the MRT systems without the kicking motion. The active drag values estimated using the MRT method were significantly higher than those obtained by the MAD system. The authors argued that the MAD system implied stroke mechanics constrictions because swimmers must propel themselves by pushing fixed pads underwater (Narita et al., 2018b). Thus, it seems that for this type of measurements, allowing swimmers to move as under “free swimming” conditions represents a key factor.

It must be pointed out that in the present study swimmers used equipment (both drag and propulsion) that allowed them to represent their swimming technique during competition and consequently, their swimming speed. Moreover, it was shown that CDa could be measured by different types of equipment presenting non-significant differences between measurements. In this sense, one can state that researchers, coaches, and swimmers should use the CDa as the main output to evaluate a swimmer’s hydrodynamic profile.

As the main limitations, it can be considered that the propulsion method: (i) relies on a differential pressure system (however, no gold-standard method exists to measure propulsion), and; (ii) measures only the propulsion of the upper limbs. However, one must claim that research has confirmed that the propulsion of the upper limbs accounts for nearly 90% of the swimming velocity (Bartolomeu et al., 2018; Deschodt et al., 1999).

Future studies should rely on: (1) verifying the CD_a_ agreement between other equipment, i.e., drag and/or propulsion, to highlight the CDa as the main hydrodynamic output in swimming, and (2) confirming these assumptions regarding the passive drag coefficient.

## Conclusions

It can be concluded that the CDa can be measured through drag and propulsion methods. Non-significant differences with a high level of agreement were found in the CDa measured with both systems. Based on this level of agreement, one can consider that the CDa should be the output taken into consideration to understand the swimmers’ hydrodynamic profile while swimming. Thus, the swimming community is advised to include the CDa whenever assessing active drag in swimming.

## References

[ref1] Alcock, A., and Mason, B. (2007). Biomechanical analysis of active drag in swimming. In A. Alcock and B. Mason (Eds.), Conference Proceedings of the 25th International Society of Biomechanics in Sports (pp. 212–215). ISBS, Ouro Preto, Brazil.

[ref2] Barbosa, T. M., Yam, J. W., Lum, D., Balasekaran, G., and Marinho, D. A. (2020). Arm-pull thrust in human swimming and the effect of post-activation potentiation. Scientific Reports, 10, 8464. 32440004 10.1038/s41598-020-65494-z PMC7242395

[ref3] Barbosa, T. M., Morais, J. E., Forte, P., Neiva, H., Garrido, N. D., and Marinho, D. A. (2017). Correction: A comparison of experimental and analytical procedures to measure passive drag in human swimming. PLoS ONE, 10(7), 0177038. 10.1371/journal.pone.0130868 PMC4514895 26207364

[ref4] Barbosa, T. M., Morais, J. E., Marques, M. C., Silva, A. J., Marinho, D. A., and Kee, Y. H. (2015). Hydrodynamic profile of young swimmers: changes over a competitive season. Scandinavian Journal of Medicine and Science in Sports, 25(2), 184–196. 24975756 10.1111/sms.12281

[ref5] Bartolomeu, R. F., Costa, M. J., and Barbosa, T. M. (2018). Contribution of limbs’ actions to the four competitive swimming strokes: a nonlinear approach. Journal of Sports Sciences, 36(16), 1836–1845. 29318954 10.1080/02640414.2018.1423608

[ref6] Bland, J. M., and Altman, D. (1986). Statistical methods for assessing agreement between two methods of clinical measurement. The Lancet, 327(8476), 307–310. 2868172

[ref7] Deschodt, V. J., Arsac, L. M., and Rouard, A. H. (1999). Relative contribution of arms and legs in humans to propulsion in 25-m sprint front-crawl swimming. European Journal of Applied Physiology and Occupational Physiology, 80(3), 192–199. 10453920 10.1007/s004210050581

[ref8] Formosa, D. P., Toussaint, H. M., Mason, B. R., and Burkett, B. (2012). Comparative analysis of active drag using the MAD system and an assisted towing method in front crawl swimming. Journal of Applied Biomechanics, 28(6), 746–750. 22695220 10.1123/jab.28.6.746

[ref9] Gourgoulis, V., Aggeloussis, N., Mavridis, G., Boli, A., Kasimatis, P., Vezos, N., ... and Mavrommatis, G. (2013). Acute effect of front crawl sprint resisted swimming on the propulsive forces of the hand. Journal of Applied Biomechanics, 29(1), 98–104. 22813753 10.1123/jab.29.1.98

[ref10] Hazrati, P., Sinclair, P. J., Ferdinands, R. E., and Mason, B. R. (2016). Reliability of estimating active drag in swimming using the assisted towing method with fluctuating speed. Sports Biomechanics, 15(3), 283–294. 27126742 10.1080/14763141.2016.1161064

[ref11] Havriluk, R. (2013). Seasonal variations in swimming force and training adaptation. Journal of Swimming Research, 21(1), 1–8.

[ref12] Havriluk, R. (2003). Performance level differences in swimming drag coefficient. In 7th Olympic World Congress on Sport Sciences: Physical, Nutritional and Psychological Care of the Athlete in the 21^st^ Century: Book of Abstracts. IOC Medical Commission, Athens, Greece.

[ref13] Hollander, A. P., De Groot, G., van Ingen Schenau, G. J., Toussaint, H. M., De Best, H., Peeters, W., ... and Schreurs, A. W. (1986). Measurement of active drag during crawl arm stroke swimming. Journal of Sports Sciences, 4(1), 21–30. 3735480 10.1080/02640418608732094

[ref14] Hopkins, W.G. (2002). A scale of magnitude for effect statistics. In A New View of Statistics (pp. 50). Will G. Hopkins, Melbourne, Australia.

[ref15] Katz, J. (2021). Aerodynamics in motorsports. Proceedings of the Institution of Mechanical Engineers, Part P: Journal of Sports Engineering and Technology, 235(4), 324–338.

[ref16] Koga, D., Gonjo, T., Kawai, E., Tsunokawa, T., Sakai, S., Sengoku, Y., ... and Takagi, H. (2020). Effects of exceeding stroke frequency of maximal effort on hand kinematics and hand propulsive force in front crawl. Sports Biomechanics, [Epub ahead of print]. 10.1080/14763141.2020.1814852 32990171

[ref17] Kolmogorov, S. V., and Duplishcheva, O. A. (1992). Active drag, useful mechanical power output and hydrodynamic force coefficient in different swimming strokes at maximal velocity. Journal of Biomechanics, 25(3), 311–318. 1564064 10.1016/0021-9290(92)90028-y

[ref18] Koo, T. K., and Li, M. Y. (2016). A guideline of selecting and reporting intraclass correlation coefficients for reliability research. Journal of Chiropractic Medicine, 15(2), 155–163. 27330520 10.1016/j.jcm.2016.02.012 PMC4913118

[ref19] Lanotte, N., Annino, G., Bifaretti, S., Gatta, G., Romagnoli, C., Salvucci, A., and Bonaiuto, V. (2018). A new device for propulsion analysis in swimming. Multidisciplinary Digital Publishing Institute Proceedings, 2(6), 285.

[ref20] Malizia, F., and Blocken, B. (2021). Cyclist aerodynamics through time: Better, faster, stronger. Journal of Wind Engineering and Industrial Aerodynamics, 214, 104673.

[ref21] Marinho, D. A., Barbosa, T. M., Costa, M. J., Figueiredo, C., Reis, V. M., Silva, A. J., and Marques, M. C. (2010). Can 8-weeks of training affect active drag in young swimmers?. Journal of Sports Science and Medicine, 9(1), 71–78. 24149388 PMC3737960

[ref22] Mason, B., Sacilotto, G., and Menzies, T. (2011). Estimation of active drag using an assisted tow of higher than max swim velocity that allows fluctuating velocity and varying tow force. Portuguese Journal of Sport Sciences, 11(2), 327–330.

[ref23] McKay, A. K., Stellingwerff, T., Smith, E. S., Martin, D. T., Mujika, I., Goosey-Tolfrey, V. L., ... and Burke, L. M. (2022). Defining training and performance caliber: a participant classification framework. International Journal of Sports Physiology and Performance, 17(2), 317–331. 34965513 10.1123/ijspp.2021-0451

[ref24] Morais, J. E., Sanders, R. H., Papic, C., Barbosa, T. M., and Marinho, D. A. (2020a). The influence of the frontal surface area and swim velocity variation in front crawl active drag. Medicine and Science in Sports and Exercise, 52(11), 2357–2364. 33064409 10.1249/MSS.0000000000002400

[ref25] Morais, J. E., Forte, P., Nevill, A. M., Barbosa, T. M., and Marinho, D. A. (2020b). Upper-limb kinematics and kinetics imbalances in the determinants of front-crawl swimming at maximal speed in young international level swimmers. Scientific Reports, 10, 11683. 32669605 10.1038/s41598-020-68581-3 PMC7363921

[ref26] Morais, J. E., Marinho, D. A., Arellano, R., and Barbosa, T. M. (2019). Start and turn performances of elite sprinters at the 2016 European Championships in swimming. Sports Biomechanics, 18(1), 100–114. 29578384 10.1080/14763141.2018.1435713

[ref27] Morais, J. E., Marques, M. C., Marinho, D. A., Silva, A. J., and Barbosa, T. M. (2014). Longitudinal modeling in sports: Young swimmers’ performance and biomechanics profile. Human Movement Science, 37, 111–122. 25150801 10.1016/j.humov.2014.07.005

[ref28] Narita, K., Nakashima, M., and Takagi, H. (2017). Developing a methodology for estimating the drag in front-crawl swimming at various velocities. Journal of Biomechanics, 54, 123–128. 28249682 10.1016/j.jbiomech.2017.01.037

[ref29] Narita, K., Nakashima, M., and Takagi, H. (2018a). Effect of leg kick on active drag in front-crawl swimming: Comparison of whole stroke and arms-only stroke during front-crawl and the streamlined position. Journal of Biomechanics, 76, 197–203. 29921521 10.1016/j.jbiomech.2018.05.027

[ref30] Narita, K., Ogita, F., Nakashima, M., and Takagi, H. (2018b). Comparison of active drag using the MRT-method and the MAD-system in front crawl swimming. Multidisciplinary Digital Publishing Institute Proceedings, 2(6), 287.

[ref31] Neiva, H. P., Fernandes, R. J., Cardoso, R., Marinho, D. A., and Abraldes, J. A. (2021). Monitoring master swimmers’ performance and active drag evolution along a training mesocycle. International Journal of Environmental Research and Public Health, 18(7), 3569. 33808199 10.3390/ijerph18073569 PMC8038111

[ref32] Pendergast, D., Mollendorf, J., Zamparo, P., Termin, A 2nd., Bushnell, D., and Paschke, D. (2005). The influence of drag on human locomotion in water. Undersea Hyperbaric Medicine, 32(1), 45-57. 15796314

[ref33] Santos, C. C., Marinho, D. A., and Costa, M. J. (2022). The mechanical and efficiency constraints when swimming front crawl with the Aquanex System. Journal of Human Kinetics, [Epub ahead of print]. 10.2478/hukin-2022-0090 PMC9679191 36457477

[ref34] Toussaint, H. M., Roos, P. E., and Kolmogorov, S. (2004). The determination of drag in front crawl swimming. Journal of Biomechanics, 37(11), 1655–1663. 15388307 10.1016/j.jbiomech.2004.02.020

[ref35] Toussaint, H. M., and Beek, P. J. (1992). Biomechanics of competitive front crawl swimming. Sports Medicine, 13(1), 8–24. 1553457 10.2165/00007256-199213010-00002

[ref36] Van Houwelingen, J., Schreven, S., Smeets, J. B., Clercx, H. J., and Beek, P. J. (2017). Effective propulsion in swimming: grasping the hydrodynamics of hand and arm movements. Journal of Applied Biomechanics, 33(1), 87–100. 27705060 10.1123/jab.2016-0064

[ref37] Veiga, S., and Roig, A. (2016). Underwater and surface strategies of 200 m world level swimmers. Journal of Sports Sciences, 34(8), 766–771. 26186108 10.1080/02640414.2015.1069382

